# A green replacement route to produce phosphatidylserine in environmentally friendly edible oil–water systems and investigations on the enzymatic mechanism

**DOI:** 10.1111/1750-3841.17544

**Published:** 2024-11-26

**Authors:** Tiantian Zhang, Haizhi Lan, Huan Wang, Binglin Li, Martin Gand, Jiao Wang

**Affiliations:** ^1^ College of Petroleum and Chemical Engineering Longdong University Qingyang China; ^2^ Gansu Key Laboratory of Efficient Utilization of Oil and Gas Resources in Longdong Gansu China; ^3^ College of Food Science and Engineering Northwest University Xi'an China; ^4^ School of Chemical Engineering Northwest University Xi'an China; ^5^ Institute of Food Chemistry and Food Biotechnology, Justus Liebig University Giessen Giessen Germany; ^6^ BioQuant Heidelberg University Heidelberg Germany

**Keywords:** molecular dynamics, oil–water systems, phosphatidylserine, phospholipase D, transphosphatidylation

## Abstract

Phosphatidylserine (PS) has many applications in functional food and pharmaceutical industries because of its critical role in activating important signal transduction pathways and modulating neurotransmitter release and receptor function. Several edible oils have been used to construct oil–water systems for the efficient production of PS under mild conditions by phospholipase D (PLD)‐mediated transphosphatidylation. Toxic organic solvents were completely avoided throughout the process, from manufacturing to encapsulation. Results showed that higher enzyme selectivity and activity were found in the coconut oil–water and olive oil–water systems than in the traditional diethyl ether–water system. The maximum yields of PS in the two oil–water systems were more than 93%, whereas that in the diethyl ether–water system was only 78%. Hence, the coconut oil–water and olive oil–water systems should be the promising medium to produce PS and its microcapsules. Moreover, the enzymatic mechanism of PLD was simulated using molecular dynamics (MD) to understand diffusion channels, competitive bindings, solvent effects, structural changes, system stabilities, and product dissociation from the enzyme. MD results showed that more PLD‐substrate complexes were detected in the oil–water systems than in the diethyl ether–water system. The conformational transition states of the protein between open and closed conformations were directly affected by the solvents used, and structural changes were observed in loop I (Asp309‐Thr336). PLD in oil–water systems can not only maintain the native structure but also broaden the diffusion channels for substrates, which contributes to a higher enzymatic activity.

## 1 INTRODUCTION

Soybean dregs, the main byproducts of the soy oil extraction process, are generated annually in several hundred million tons and are currently mainly used to manufacture fertilizer and feed with very low market values (Shahbandeh, 2023). However, soybean dregs are rich in phospholipids (PLs). Among those numerous PLs, phosphatidylserine (PS) is used in several relevant processes in the human body, such as signal transduction and bone formation (Glade & Smith, 2015). In many countries, PS is used as a drug adjuvant or bioactive molecule in food to prevent and reduce the adverse effects of some nervous system diseases (Dong et al., 2023). Clinical studies have indicated that PS could cross the blood–brain barrier (Glade & Smith, 2015). Therefore, after oral administration, PS can be adsorbed efficiently and regulate biochemical alterations and structural deterioration in neuronal cells (Glade & Smith, 2015). However, the low content of PS in soybean dregs (<1 %) limits its application. The major component of soybean dreg‐derived lecithin is phosphatidylcholine (PC; up to 30%), but its production is overcapacitated due to its very low physiological activity (Lin et al., 2021; D. E. Vance, 1989). Hence, if PC can be converted to PS, it will not only solve the overcapacity problem of soybean PLs but will also significantly increase the added value of soybean processing.

The widely applied strategy for the production of naturally rare PLs is the phospholipase D (PLD)‐catalyzed transphosphatidylation (Damnjanović & Iwasaki, 2013). Due to the poor solubility of PLs in water, transphosphatidylation is generally performed in a biphasic system consisting of an organic phase (e.g., chloroform, diethyl ether, and ethyl acetate) and an aqueous phase (Damnjanović & Iwasaki, 2013). The biphasic system has the drawback of unsatisfactory yields, accumulation of toxic solvents, and complicated separation processes. Toxic organic solvents (TOSs) should be minimized or avoided during PS production. Hence, some expensive less‐toxic solvents were used to build micro aqueous or anhydrous reaction systems for transphosphatidylation, such as ionic liquids, γ‐valerolactone, and deep eutectic solvents (Bi et al., 2015; Duan & Hu, 2012; Pinsolle et al., 2013). However, enzyme denaturation may be a limitation for these systems.

We constructed several aqueous–solid systems in which PC was dispersed on existing carriers by precipitation or adsorption (Li et al., 2018, 2019, 2016; Wang et al., 2021; X. Zhang et al., 2017). Although the amount of volatile organic compounds was reduced or avoided in the production process, organic solvents were still required to extract the final PS from the solid phases. Edible oils are natural products extracted from drupes, vegetables, or seeds with low melting, high boiling and flash points, and low vapor pressure (Castro et al., 2020; Viana da Silva et al., 2021; Yao & Xu, 2021). They are non‐toxic, renewable, and biodegradable. Owing to their attractive physical and chemical properties, edible oils are excellent candidates as green solvents for preparing PS.

In this study, novel food‐grade oil–water systems were designed to produce PS efficiently under mild conditions by PLD‐mediated transphosphatidylation. Reaction parameters, including different edible oils, operational temperature, pH, molar ratio, and solvent ratio, were systematically investigated in the present study. Moreover, molecular dynamics (MD) simulations were employed to reveal the enzymatic mechanism of PLD in different reaction systems. All enzymatic processes were reconstructed quantitatively in silico to analyze their kinetic behavior and investigate the structure–activity relationship of proteins in different systems.

## 2 MATERIALS AND METHODS

### 2.1 Chemicals and enzymes

PLD (*Streptomyces* sp.) and soybean PC, PS, and phosphatidic acid (PA) were obtained from Merck KGaA. PLD was stored at 4°C in acetate buffer (pH 5.5) with a diluted concentration of 3.10 × 10^−4 ^g protein/mL. Refined and virgin coconut oils were provided by Zhi Zhi Su Biological Resources Co. Various oils were purchased from different distributors: refined and virgin olive oil from Olivoila Co., peanut and canola oil from Shandong Luhua Group Co., Ltd., and soybean oil from Jin Long Yu Co., Ltd. l‐Serine was obtained from Aladdin Ind. Co.

### 2.2 Production of PS

Transphosphatidylation was performed in different edible oil–water systems. Ten milligrams of PC was dissolved in 2 mL of the edible oil, followed by the addition of 1 mL of acetate buffer (0.2 M, pH 5.5) containing 140 mg of l‑serine. Then, 10 µL of PLD solution was added to start the reaction, which was performed at 33°C and 600 rpm for 2 h. After the reaction, decompression–evaporation was performed in a vacuum at 45°C to remove all water. Meanwhile, water‐soluble impurities (including PLD, the byproduct choline, and excess l‑serine) were easily separated by centrifugation (4000 rpm, 10 min, 20°C) to obtain clear oil solutions of PLs.

To evaluate the enzymatic performance of different systems, transphosphatidylation was performed in different temperatures from 20 to 50°C. Additionally, the pH was changed from 4.5 to 7.0; acetate buffer solutions were used for changing the pH from 4.5 to 6.0, and phosphate buffer solutions were used for changing the pH from 6.5 to 7.0. The amount of l‐serine was changed to adjust the molar ratio of l‐serine to PC from 50 to 150 (70–210 mg of l‐serine). The effect of the oil content was investigated by altering the volume of the oil phase in the volume ratio of organic to aqueous phases from 0.5 to 4.

### 2.3 Chemical analysis of PLs

For edible oil–water systems, the product PLs should be separated to improve analysis accuracy because triglycerides have similar physicochemical properties to PLs, which exert a negative effect on separation during high‐performance liquid chromatography (HPLC). After transphosphatidylation, oil solutions (0.5 mL) of PLs were mixed with hexane (2.5 mL) followed by the addition of 100 mg Al_2_O_3_ to adsorb PLs. The Al_2_O_3_‐adsorbed PLs were washed with cooled acetone to remove residual oil. Next, PLs were extracted using a diethyl ether/methanol mixture (1:2, v/v) and analyzed using the Shimadzu LC‐20AT HPLC system equipped with an ultraviolet detector at 204 nm. Other parameters were the same as those used in a previous study (Wang et al., 2019). Additionally, ^31^P NMR (Avance III; Bruker) was employed to further analyze the components of PLs according to the reported method by Y. Zhang et al. (2022).

### 2.4 Comparison of the reaction systems

For comparison, transphosphatidylation was performed using a traditional diethyl ether–water system. A mixture consisting of 2 mL of diethyl ether including 10 mg of PC, 1 mL of 0.2 M acetate buffer (pH 5.5), including 140 mg of l‑serine, and 10 µL of PLD solution, was used for the reaction at 30°C and 600 rpm for 2 h. After the reaction, decompression–evaporation was performed in a vacuum under 45°C to remove all solvents. The mixture (2:1, diethyl ether/methanol, v/v) was used to extract PLs from the precipitates. The collected solutions were evaporated under a vacuum under 45°C. Finally, the obtained precipitates were further extracted using 2 mL of diethyl ether and analyzed by HPLC, as described in Section 2.3.

### 2.5 Protein modeling

PLD from *Streptomyces cinnamoneus* (GenBank ID: BAA75216) is an enzyme with high transphosphatidylation activity and a protein sequence that represents the commercial *Streptomyces* sp. PLD enzyme (Ogino et al., 2008). Alphafold2 was used to build the protein structure model because the known crystal structures of PLD enzymes mainly exhibit hydrolytic activity and not transphosphatidylation activity (Jumper et al., 2021). The obtained PLD structure was placed into an aqueous microunit (91 × 91 × 91 Å^3^) for further MD analysis using the NAMD v.2.14 software to generate a relaxed and reliable structure model (Phillips et al., 2002). The detailed experimental method can be found in the reconstruction of the microunits of the reaction systems in silico in Section 2.6. The structure model of PLD was considered when the value of root‐mean‐square deviation (RMSD) of the whole carbon skeleton of PLD was in the equilibrium state with the fluctuation below 0.5 Å (data not shown).

### 2.6 Reconstruction of microunits of reaction systems in silico

The structures of the PC, l‐serine, and water molecules were obtained from the Ligand Expo database of Protein Data Bank. Fatty acids were downloaded from the CHARMM‐GUI database (Lee et al., 2015; Wu et al., 2014). Three microunits (coconut oil–water, olive oil–water, and diethyl ether–water systems) were built using Packmol v.20.1.0 software (Martínez et al., 2009) based on experimental conditions as follows: the volume ratio of organic/aqueous phases was 2:1 and the temperature and pressure were set as 30°C and 1 atm, respectively. The size of each micronutrient reached 90 × 90 × 108 Å^3^. The aqueous solution consisted of 7025 water molecules, 1 PLD molecule, and 100 l‐serine molecules. The organic phase contained 20 PC molecules. For the diethyl ether–water system, the number of diethyl ether molecules was 2300. For edible oil–water systems, the number of fatty acids in microunits was calculated based on Figure S1. To maximize the reaction efficiency and minimize the mass transfer resistance, all microunits were initially set as water‐in‐oil (W/O) models (Figure S1E).

NAMD software v.2.14 was used for the MD simulations based on the standard CHARMM36 force field. In all cases, the temperature and pressure were set at 30°C and 1 atm, respectively. An 11 Å of non‐bonded interaction cut‐off, 2 ps of intervals, the particle mesh Ewald method, the SHAKE algorithm, and periodic boundary conditions were performed during the whole simulation (Kalé et al., 1999; Lee et al., 2015; Phillips et al., 2005, 2002). The minimization, equilibrium, and dynamic simulations were set to 0.5, 4, and 200 ns, respectively. Other parameters were set to default values. Each micronutrient was simulated five times simultaneously. VMD v.1.9.3 software was used to analyze MD data (Humphrey et al., 1996).

### 2.7 Molecular docking

AutoDock Vina v.1.2.0 and AutoDock Tools v.1.5.6 software were used for docking based on the semi‐flexible docking method, and other parameters can be found in our previous work (Wang et al., 2021).

## 3 RESULTS AND DISCUSSION

### 3.1 Reaction parameters that affect the performance of transphosphatidylation

**FIGURE 1:**
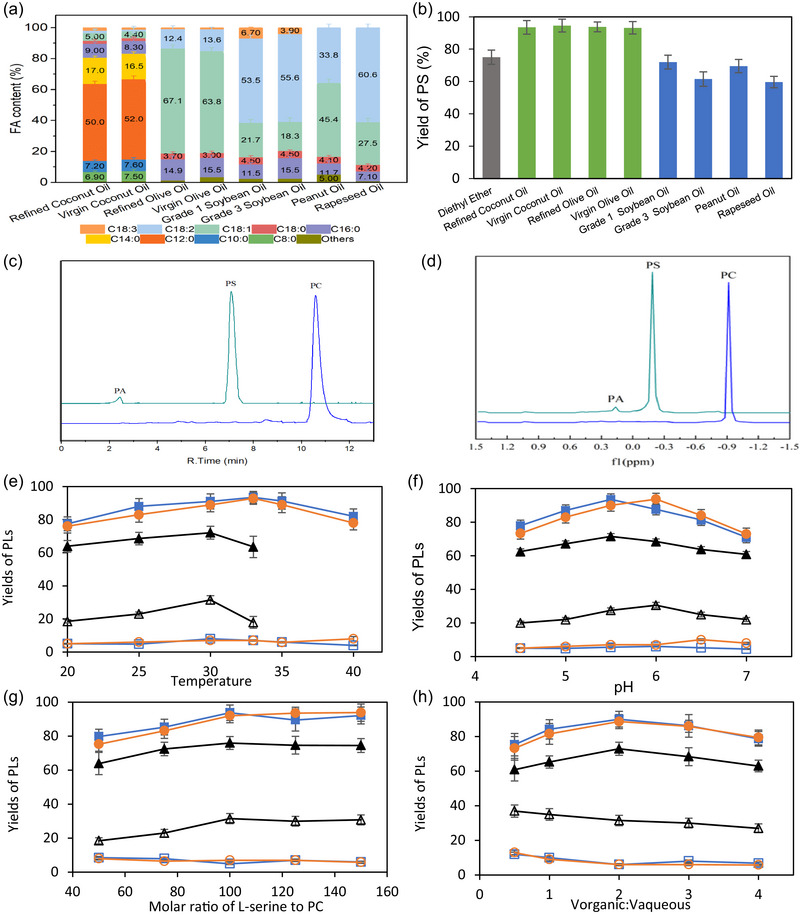
Fatty acid (FA) content of the different oils (a) and the yield of phosphatidylserine (PS) after transphosphatidylation reaction inside these oils, which were compared with diethyl ether (b). Grade 1 soybean oil and Grade 3 soybean oil refer to two different grades of soybean oil defined by the supplier. high‐performance liquid chromatography (HPLC) (c) and ^31^P NMR analysis (d) of PS from phosphatidylcholine (PC) and l‐serine in coconut oil–water system. Substrate PC is shown by the blue line. Product PS and byproduct PA were shown by the cyan line. Variables affecting the PS yield (e–h). Coconut oil, olive oil, and diethyl ether–water systems are represented by blue, orange, and black colors, respectively. PS and PA yields are represented by closed and open symbols.

The compositions of the eight edible oils were analytically investigated, and the results are presented in Figure 1a. All these oils and diethyl ether were used to produce PS in the oil–water or diethyl ether–water systems (Figure 1b). Maximum yields were observed in the coconut and olive oil–water systems, reaching over 93%. The PLs were characterized by performing HPLC and ^31^P NMR (Figure 1c,d). Inside the oil–water systems, the hydrolysis reaction, which occurred as a side reaction, was minimized. In contrast, lower yields (≤72%) of PS were measured in other edible oil–water systems such as soybean, peanut, and rapeseed oil–water systems, which were even lower than that in the traditional diethyl ether–water system (75%).

The main fatty acids present in commercial coconut and olive oils were lauric acid (50.0%) and oleic acid (63.8%), respectively. They are easily absorbed by the stomach and metabolized by the liver to generate ketone bodies. Ketone bodies, together with lactate, are the primary alternative fuels for the human brain, particularly during starvation. Therefore, there may be a potential synergistic effect between coconut/olive oil and PS products in the treatment of memory disorders and Alzheimer's disease (Glade & Smith, 2015; J. Vance & Steenbergen, 2005). These unique physiological functions make coconut and olive oils the best candidates for use as green solvents for transphosphatidylation.

As shown in Figure 1b, only a slight difference in the PS yield was observed between the refined and virgin coconut/olive oil–water systems. Therefore, virgin oil could be considered the best candidate because it preserved the maximum amount of functional nutrients as no heat treatment was performed to refine it. However, virgin coconut oil exhibited the highest melting point and existed in a solid aggregated state at room temperature, thus complicating the downstream process. Therefore, refined coconut oil and virgin olive oil were considered the best solvents.

Transphosphatidylation reactions were performed at various temperatures in the coconut/olive oil–water systems and compared with the diethyl ether–water system (Figure 1e–h). A slightly higher optimal temperature for transphosphatidylation was observed in these oil–water systems (33°C) compared with that in the diethyl ether–water system (30°C). When the operational temperature was increased, the internal energies increased, increasing the number of collisions of reactants and facilitating the formation of enzyme–substrate complexes (Prieto et al., 2015). However, extremely high temperatures induced enzyme denaturation. Meanwhile, the operational temperature affected system parameters, such as their stability. Diethyl ether with a very low boiling point (34.6°C) was easily evaporated, leading to the precipitation of PLs. Moreover, the yields of PS were maintained at ∼80% under harsh temperature conditions in oil–water systems, implying higher thermal tolerances or a protective effect provided by the system components for the used PLD.

Next, different reaction parameters such as pH, substrate molar ratio, and solvent volume ratio were systematically investigated. As shown in Figure 1f–h, high enzymatic efficiencies were observed in all edible oil–water systems, and the accumulation of PA in these systems was approximately four times less than that in the diethyl ether–water system.

### 3.2 Kinetic behaviors of ligands

The kinetics behaviors of ligands are determined by the experimental measurement of all reactants to obtain kinetic data for biomolecular interactions. However, a fast timescale for the resolution of a binding mechanism with atomic resolution remains difficult because of the intrinsically dynamic and volatile nature of the binding process. Therefore, MD has been used as an efficient biomolecular technology to elucidate the mechanism of PLD‐catalyzed transphosphatidylation in heterogeneous systems.

**FIGURE 2:**
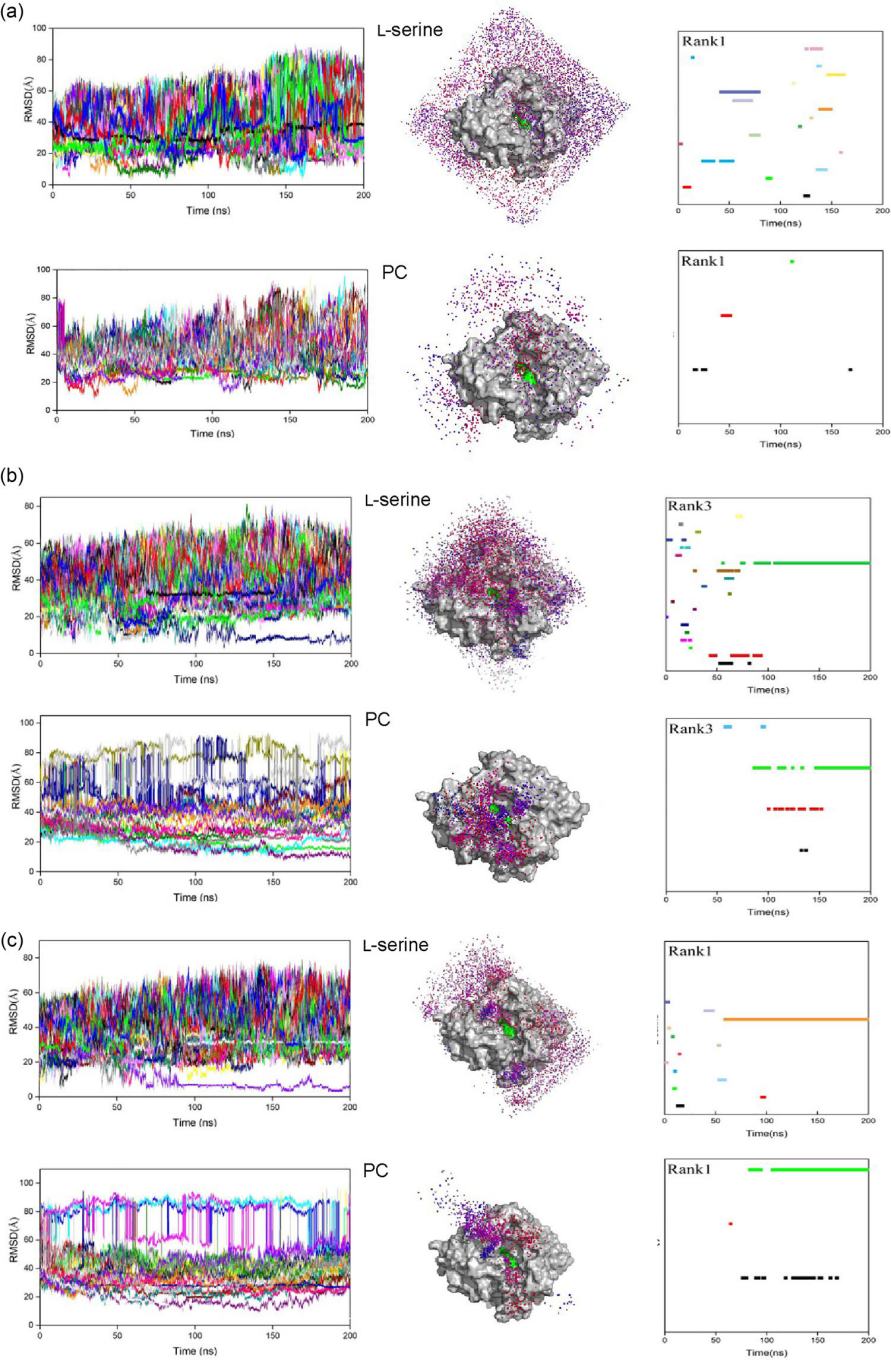
Kinetic behaviors of ligands, including the time evolution of root‐mean‐square deviation (RMSD), diffusional trajectories (only molecules bound in the active pocket were shown), and binding time of substrates (each color represents one substrate molecule) in the (a) diethyl ether; (b) the coconut; and (c) olive oil–water systems. PC, phosphatidylcholine.

**FIGURE 3:**
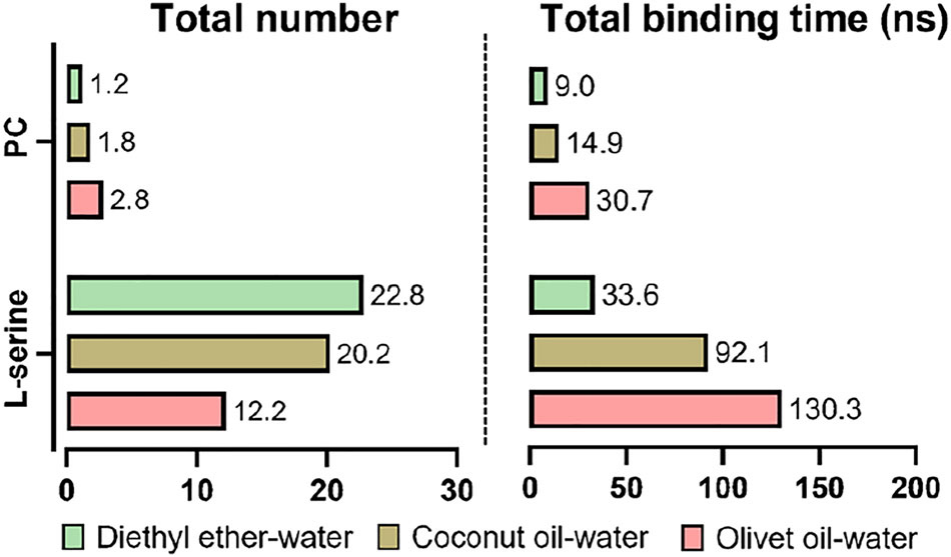
The number and total binding time of l‐serine/phosphatidylcholine (PC) entering in the active center.

RMSD of distances between l‑serine and PLD was calculated to quantitatively analyze the whole diffusion process of l‑serine. The standard for substrate binding is illustrated in Figure S2, and its description is provided in the Supporting Information. As shown in Figure 2 and Figures S3–S5, more stable PLD–serine (PLD–Ser) complexes were found only in these oil–water systems in which at least one l‑serine was bound and kept in the active pocket (RMSD < 10 Å) over 30 ns. As shown in Figure 3, the average binding time of each PLD–Ser complex was approximately three to seven times higher in these oil–water systems than in the traditional diethyl ether–water system. Although almost all PC molecules could interact with the PLD (RMSD < 35 Å), only several PC molecules entered into the active center of the PLD (RMSD < 12 Å) owing to their huge steric hindrances. The formation of the PLD–PC complex was inhibited in the two oil–oil–water systems (Figures S4 and S5). As shown in Figure S6, due to the huge number of l‑serine molecules, binding to PC is inhibited. Considering the enormous steric hindrance of PC, these molecules could only migrate around the active pocket and could not enter it. Although more PLD–PC complexes were observed in the diethyl ether–water system, they were very unstable and dissociated more easily. As shown in Figure 3, the average binding time of each PLD–PC complex was 1.1 and 1.5 times higher in coconut and olive oil–water systems than in the diethyl ether–water system, respectively. The trajectories of l‐serine and PC molecules on the PLD surface were visualized to investigate the binding pathways and kinetic behaviors of the substrates (Figure 2; Figures S3–S5). The trajectories of l‑serine and PC for both showed random scattering throughout the whole reaction microunit, and their densities were significantly lower in the active center in the diethyl ether–water system. In contrast, obvious aggregations were observed on the surface and in the active pocket of PLD when oil–water systems were used, indicating stronger enzymatic performance. As shown in Figure 2 and Figures S3–S5, the binding time profiles for each molecule were analyzed. The formation of the PLD–Ser–PC complex was evaluated by analyzing the overlap of the binding time coordinates between PC and l‐serine molecules. Unfortunately, PLD–PC complexes formed in the diethyl ether–water system were hard to further interact with l‑serine and form the PLD–Ser–PC complex, implying a higher probability for the hydrolysis reaction but not transphosphatidylation. PLD–PC complexes were always present in the PLD–Ser–PC complexes in these oil–water systems. Water molecules also acted as nucleophiles in the PLD–PC complex and caused the hydrolysis of PLs. As shown in Figure S7, there was no obvious difference in the number of water molecules among the three reaction systems, which was ∼35 in all cases. Considering the obvious differences in reaction selectivity among the three systems (Figure 1), the transphosphatidylation specificity of PLD was controlled mainly by the efficient binding of l‐serine. These phenomena suggested that the substrates were bound in the following order in these oil–water systems: PLD bound with l‐serine first, and then the PLD–Ser complex bound with PC. In contrast, substrate binding was relatively random in the diethyl ether–water system. However, the preformation of PLD–PC complexes without the l‑serine ligand probably accelerated the hydrolysis of PLs, leading to the accumulation of PA, which was consistent with experimental data (Figure 1).

After transphosphatidylation, the generated PS molecule left the active pocket to start the next cycle. Figure S8 illustrates the stability of the PLD–PS complexes. The complete dissociation of the PLD–PS complex (RMSD > 12 Å) was observed in the in silico experiments only in one case, where the diethyl ether–water system was used, whereas the fastest dissociation needed at least 13 ns. In contrast, the longest stable time for the PLD–PS complex was not over 9 ns in all oil–water systems, implying better performance.

### 3.3 Kinetic behaviors of organic solvents

**FIGURE 4:**
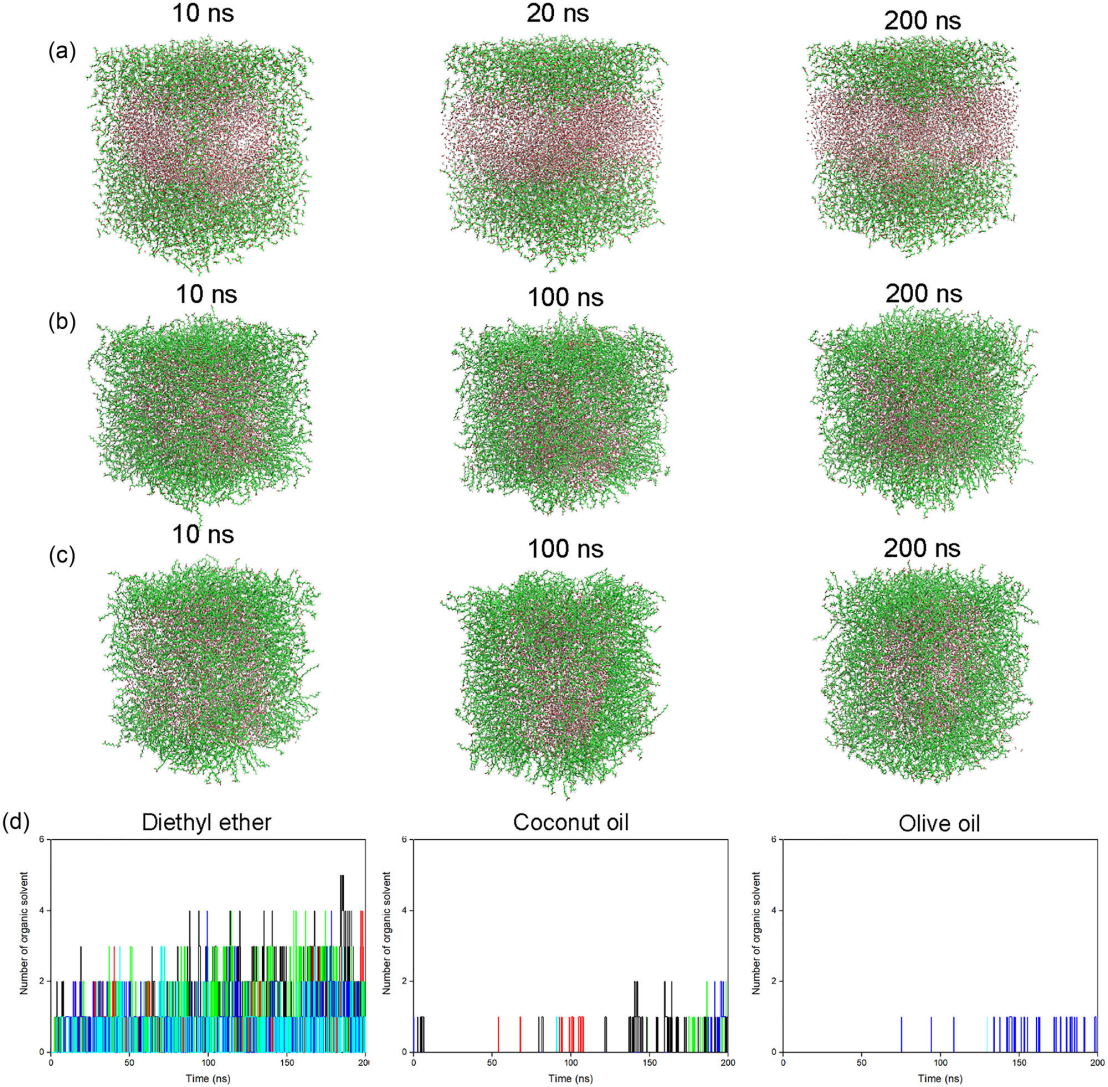
The biphasic distribution of three systems to evaluate their stabilities. (a) Diethyl ether–water system, (b) coconut oil–water system, and (c) olive oil–water system. Expect solvents, other molecules were for an easier visualization. Organic and water molecules were shown by green and red, respectively. The molecular dynamics (MD) time for each snapshot was also described. (d) Statistics of organic molecules within 10 Å from the active pocket of PLD.

As shown in Figure S1, the initial three reaction microunits were artificially set as the W/O model to maximize the mass transfer efficiency and analyze their stabilities. Snapshots were selected from the three biphasic systems to show the distribution of the molecules (Figure 4a–c). In the diethyl ether–water system, the ideal unit broke rapidly, and a clear layered structure was observed after 20 ns of MD simulation. However, the oil–water systems maintained stable initial structures throughout the process. Notably, the total number of organic solvents in the active pocket of PLD was significantly different among the three systems (Figure 4d). Oil molecules were barely detected in the active pocket and substrate tunnels of PLD, as they hardly diffused into the active pocket. Even if they entered, they were very unstable because of steric hindrance. Thus, the natural structure of the active pocket of PLD was probably well preserved in these oil–water systems. In contrast, diethyl ether, a strong hydrophobic molecule with a small size, was attracted to the hydrophobic core of the PLD. This increased the repulsion for the binding of polar l‐serine molecules. PC is a large amphiphilic molecule. The main mass‐transfer resistance existed in the hydrophobic tails. The polar head group of PC, the choline group, was first attracted by the hydrophilic outer surface of PLD, which further facilitated the adsorption of PC molecules onto the surface of the proteins (Buch et al., 2011; Wang et al., 2021). The pre‐existing diethyl ether repelled the hydrophilic choline group and thus prevented PC adsorption. This could explain why fewer PLD–Ser–PC complexes were detected in the diethyl ether–water system.

### 3.4 Kinetic behaviors of PLD

As shown in Figure 5, structural changes in PLD were analyzed to explain the enzymatic differences among the three systems. Only high‐affinity residues (HARs) are shown in different colors for easier visualization, which does not mean that there is no affinity for the substrate in the black‐colored region(s). Barely any changes were observed in the marine blue or slate gray regions indicating that HARs were almost located on high flexible regions (RMSD > 4 Å). Meanwhile, the two substrates barely shared HARs. This may be caused by the steric resistance of PC. When PLD was used in the diethyl ether–water system, HARs were further away from the active center, and a lower brightness was observed in the clustered heatmap compared with that in oil–water systems. Based on the diffusion trajectories of the substrates (Figure 2 and Figures S3–S5), three important loops were confirmed (I, Asp309‐Thr336; II, Ala372‐Ala‐378; and III, la406‐Ser417). As shown in Figure S9, loops I–III, together with the stable loop IV (Ser196‐Asn200+Gly154‐Met161), consisted of the binding channel and controlled the diffusion of substrates. The most significant difference was observed in loop I, which was unfolded and exposed to the protein exterior in the diethyl ether–water system. This change prevented the diffusion of the substrates, which were adsorbed on the back side and diffused by parallel movement. This may also explain why HARs were barely detected in loop I (Figure 5a). In contrast, loop I was stable in both oil–water systems, and fluctuations were only observed in the end part from Lys327 to Thr336. These changes increased the distance between loops I and IV, resulting in a wider diffusion channel. Diethyl ether exerted a negative effect on enzymatic activity because all diffusion channels were narrower. Loop II was gradually unfolded and compressed the space with loop III in the diethyl ether–water system, but it was very stable in oil–water systems and presented as the folded status. Although loop III was also unfolded slightly, it refolded soon in oil–water systems. Therefore, the higher enzymatic activity could be attributed to the protective effect exerted by these oil–water systems, which could help PLD maintain its native structure and even improve its diffusion channels.

FIGURE 5The schematic consists of residual root‐mean‐square deviation (RMSD), cluster analysis of each residue with l‐serine (upper)/ phosphatidylcholine (PC) (lower), enzymatic changes shown by cartoon (transparent: initial structure; palecyan: high flexible regions [HFRs]; red: more flexible loops I–III) and surface (black: stable regions; marine blue: high affinity residues [HARs] of l‐serine; slate gray: HARs of PC; pink: HARs of l‐serine in HFRs; violet: HARs of pc in HFRs; magentas: HARs of both substrates in HFRs;), and distances in (a) diethyl ether; (b) coconut oil; (c) olive oil–water systems. Average values were used basing on five molecular dynamics (MD) cases.

**Figure:**
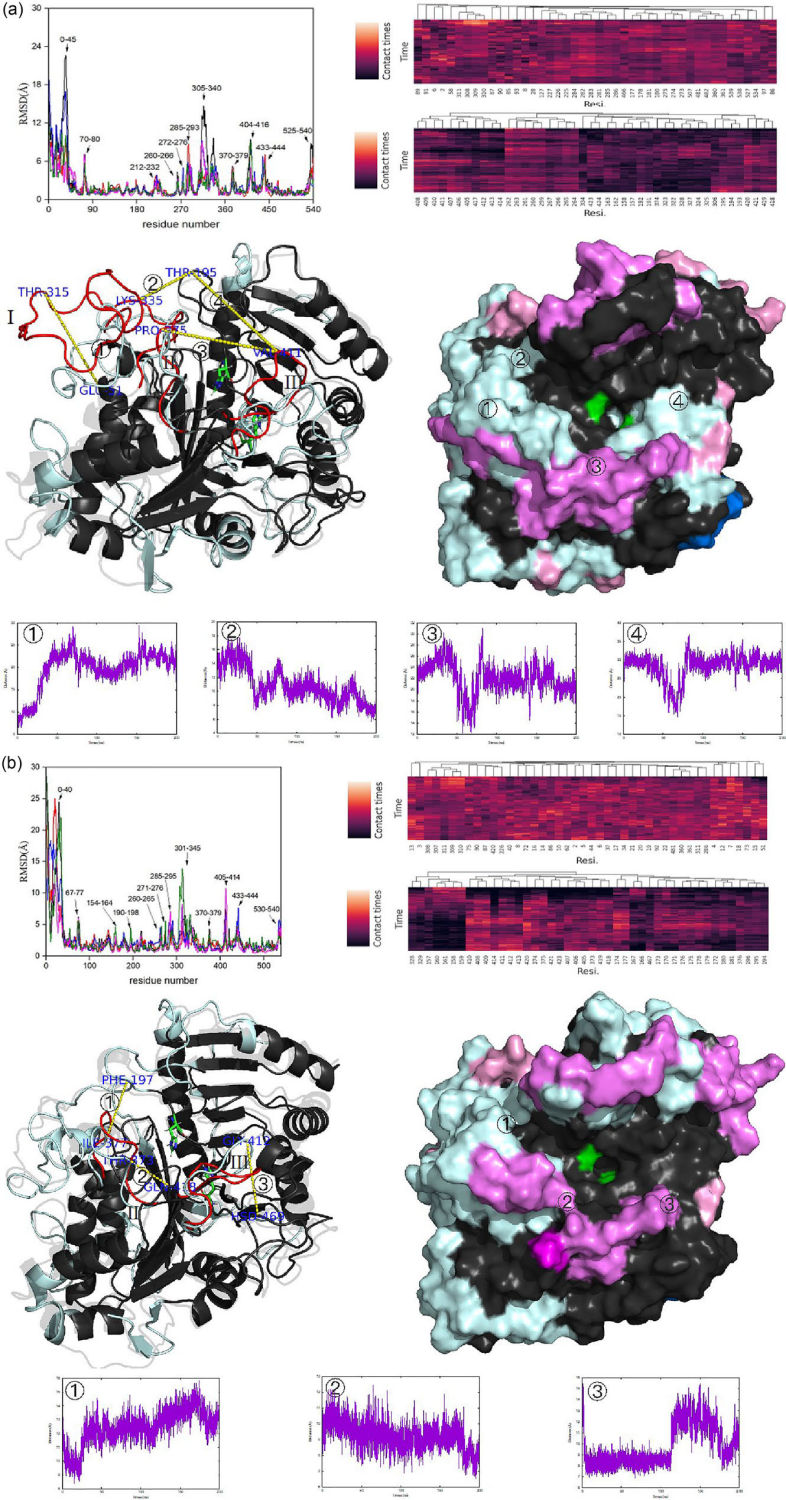

**Figure:**
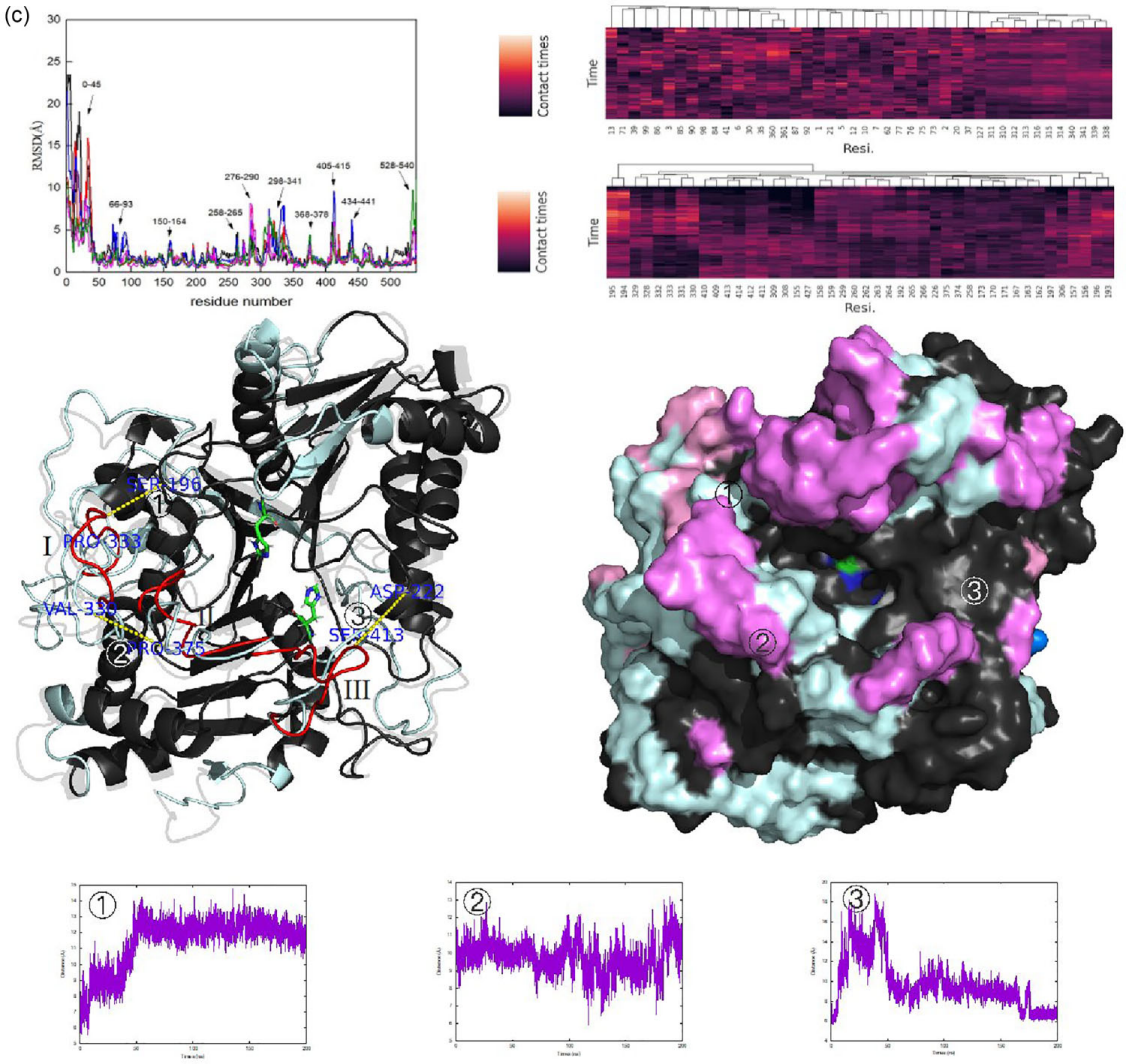

## 4 CONCLUSION

Novel and efficient food‐grade oil–water systems were constructed and successfully used for the first time to produce PS with high purity (≥93%). After transphosphatidylation, the direct formulation of the microcapsules was investigated (Figure S10). Compared with recent studies (Table S1), herein, TOSs were completely avoided during the entire process, from production to packaging. This method provides a promising way to produce PS and its microcapsules in an environmentally friendly system. Moreover, heterogeneous reaction microunits for PLD were constructed and analyzed in silico using MD simulations. The dynamic behaviors and relevant changes in the enzymatic performance of PLD used in different reaction systems were studied, which can explain the experiment performances at the molecular level.

## AUTHOR CONTRIBUTIONS

**Tiantian Zhang**: Writing—review and editing; writing—original draft; investigation; software; data curation; formal analysis; funding acquisition; visualization. **Haizhi Lan**: Investigation. **Huan Wang**: Investigation. **Binglin Li**: Supervision; conceptualization; methodology; project administration; funding acquisition. **Martin Gand**: Methodology; project administration. **Jiao Wang**: Conceptualization; methodology; project administration; supervision; validation.

## CONFLICT OF INTEREST STATEMENT

The authors declare no conflict of interest.

## Supporting information



Supplementary materials

## Data Availability

The data that support the findings of this study are available from the corresponding author upon reasonable request.
