# Supplementary material for: A green replacement route to produce phosphatidylserine in environmentally friendly edible oil–water systems and investigations on the enzymatic mechanism
Source: J Food Sci. 2024 Nov 26;89(12):9154–65. doi: 10.1111/1750-3841.17544 (PMC11673409; doi:10.1111/1750-3841.17544)
Supplement: Supplementary file 1 — Supplementary materials [file JFDS-89-9154-s001.docx]

**A green replacement route to produce phosphatidylserine in environmentally friendly edible oil–water systems and investigations on the enzymatic mechanism**

Tiantian Zhang^1,2^, Haizhi Lan^3^, Huan Wang^4^, Binglin Li^3^, Martin Gand^5^*, Jiao Wang^3,6^*

1 College of Petroleum and Chemical Engineering, Longdong University, Qingyang, Gansu 745000, China

2. Gansu Key Laboratory of Efficient Utilization of Oil and Gas Resources in Longdong, Longdong University, Qingyang, Gansu 745000, China

3 College of Food Science and Engineering, Northwest University, Xi’an, Shaanxi 710000, China

4 School of Chemical Engineering, Northwest University, Xi'an, Shaanxi 710000, China

5 Institute of Food Chemistry and Food Biotechnology, Justus Liebig University Giessen, Giessen 35392, Germany

6 BioQuant, Heidelberg University, Heidelberg 69120, Germany

Corresponding Author:

Martin Gand

Email: martin.gand@lcb.chemie.uni-giessen.de

Jiao Wang

Email: jiao.wang@bioquant.uni-heidelberg.de

**Table S1.** Comparison of several reaction systems for PS synthesis. VOCs: volatile organic solvents.

|  | Yield of PS [%] | Use of VOCs | Additional surfactants | Potential source of contamination | Purification | Effluent | Safety | Cost |
| --- | --- | --- | --- | --- | --- | --- | --- | --- |
| Coconut/olive oil-water system | 95.9 | No | No | __ | Decompression-evaporation | Water | High | Cheap |
| Immobilized PLD in aqueous system | 95.3 | Yes^b^ | No | __ |  |  | High | Medium |
| Diethyl ether-water system | 76.9 | Yes^c^ | No | Solvents, PLD, l‑serine, choline | Extraction, column chromatography, extraction and decompression-evaporation, drying | Water, diethyl ether, chloroform, methanol, etc. | Low | Cheap |
| Less toxic solvent system*^a^* | 95 | Yes^d^ | No | l‑serine, choline | Centrifugation or filtration | __ | High | Expensive |
| Aqueous-solid system | 99 | Yes^e^ | No | Eluent, PLD | Centrifugation, elution, decompression-evaporation, drying | Water, ethanol, chloroform, methanol | High | Medium |
| Aqueous suspension system | 70.2 | Yes^d^ | No | Eluent, PLD | Centrifugation, elution, column chromatography, decompression-evaporation, drying | Water, ethanol, chloroform, methanol | Low | Expensive |
| Mixed micelles system | 57 | Yes^d^ | Yes | Surfactants, extractants | Extraction, column chromatography, decompression-evaporation, drying | Water, diethyl ether, chloroform, methanol, etc. | Low | Expensive |

^a^The γ-valerolactone system has the best performance in all reported less toxic solvent systems and was therefore selected to comparison with other systems. Organic solvents were used in ^b^immobilization of PLD; ^c^transphosphatidylation and product purification; ^d^product purification; ^e^modification of silica.

**Figure S1.** GC analysis of fatty acid compositions. (A) 37 kinds of FAME standard samples; (B) coconut oil; (C) olive oil. (D) Composition of micro-units of oil-water systems.

(D) Three-dimensional presentation of the initial W/O micro-unit.


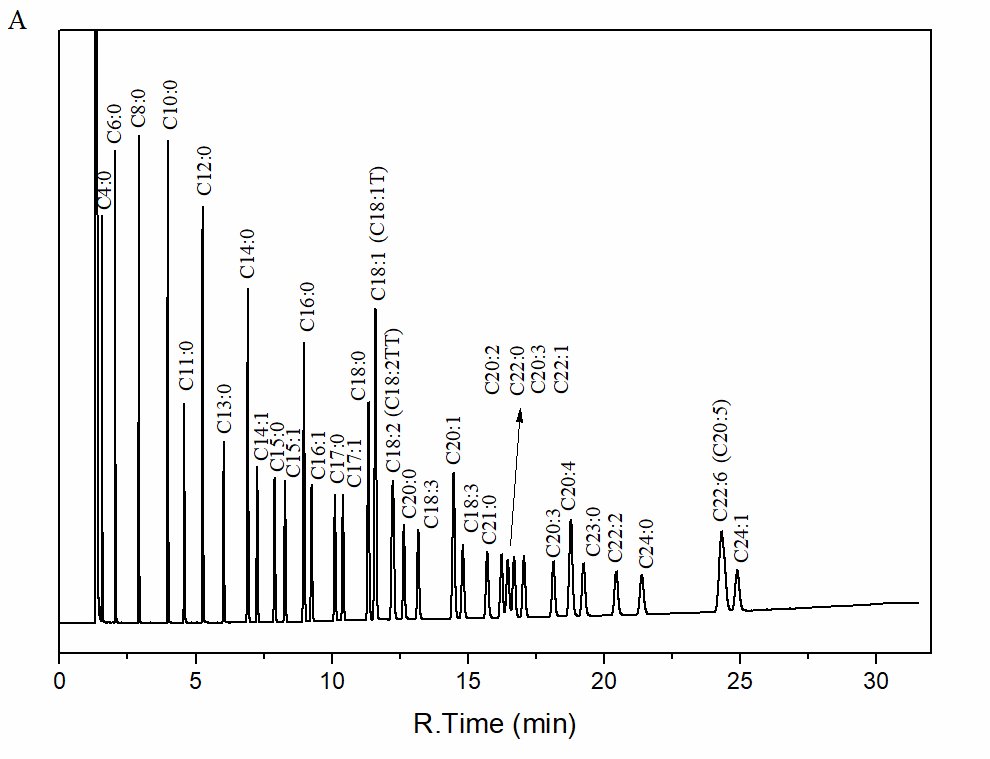

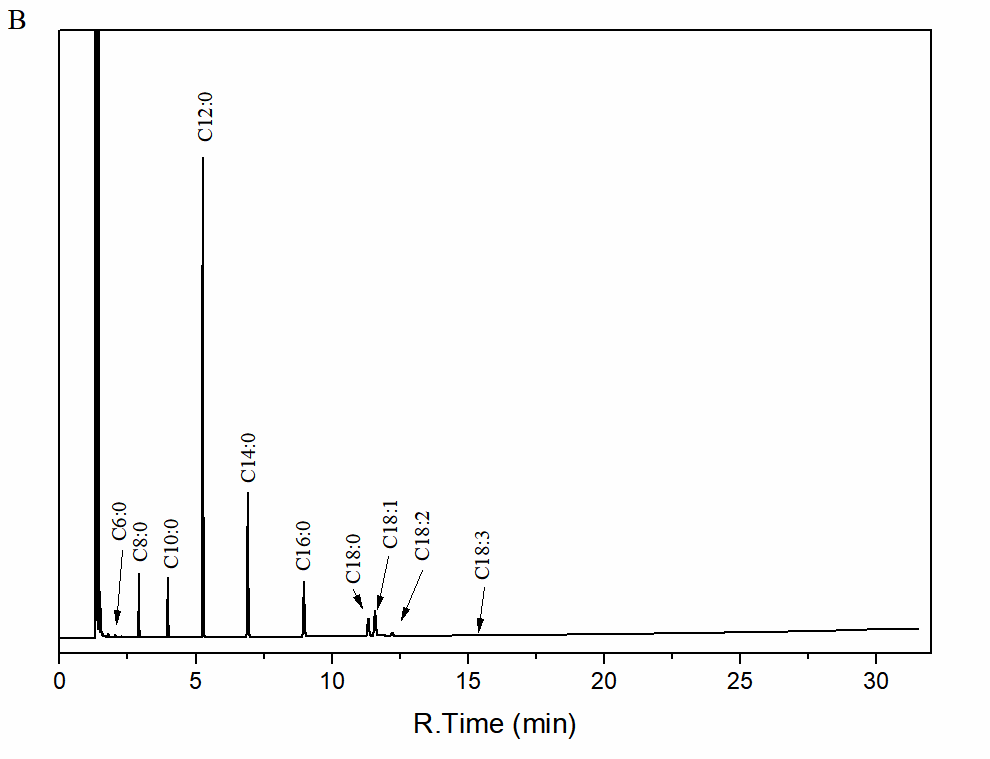

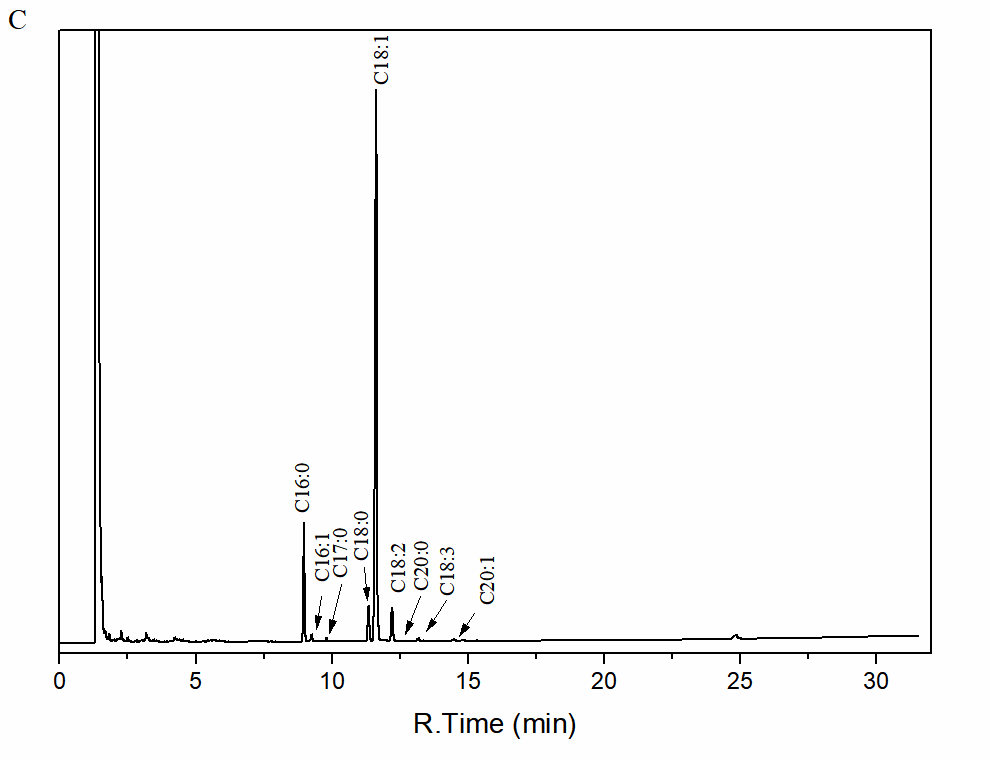

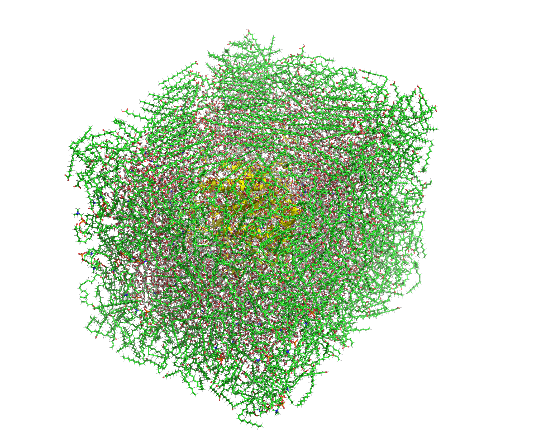


Diethyl ether-water


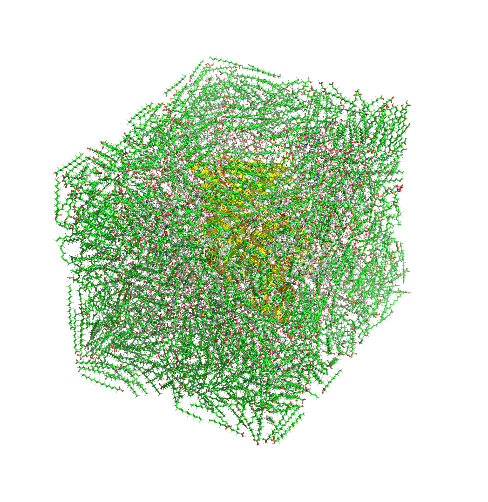


Coconut oil-water


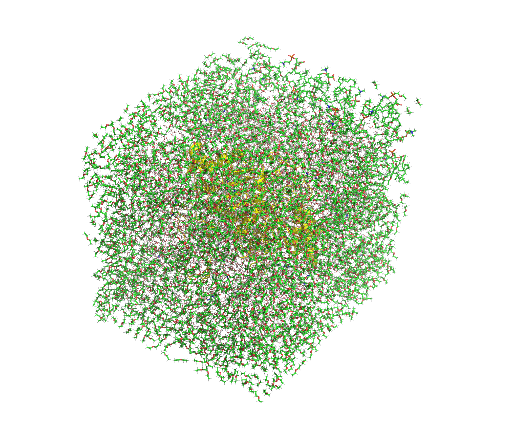


Olive oil-water

E

D

For edible oils, all triglycerides were converted to fatty acid methyl esters (FAMEs)(Zhang et al., 2022) to analyze the fatty acid compositions by GC (Gas Chromatography) using a Shimadzu GC2030 GC (Tokyo, Japan) equipped with a flame-ionization detector. The column was SH-Rtx-wax (30 m × 0.25 mm × 0.25 um, Shimadzu). The temperature of injector and detector were set to 250 °C and 280 °C, respectively. The heating program for the oven was set as following: 165 °C for 1 min, a ramp of
6.5 °C /min to 210 °C, a ramp of 1.5 °C /min to 220 °C, and kept for 1 min. The flow rates of N_2_, H_2_ and air were set to 24, 32 and 200 mL/min.

**Figure S2.** Representative PLD-Ser-PC complex structure, generated by molecular docking. The interaction between substrates and PLD was shown by 2D and 3D models, respectively. Residues in the red cycle could interact with l‑serine and PC simultaneously in the 2D model (A and B). For the 3D model (C), the active center of PLD was shown by green. PC and l‑serine were colored in orange and magenta, respectively. (D) MD stability of PLD-Ser-PC complex. The crucial distance between P atom of PC and N atom (NE2) of residue H201 in PLD (blue line); The crucial distance between O atom of the hydroxy group in l‑serine and N atom (NE2) of residue H474 in PLD (orange line);


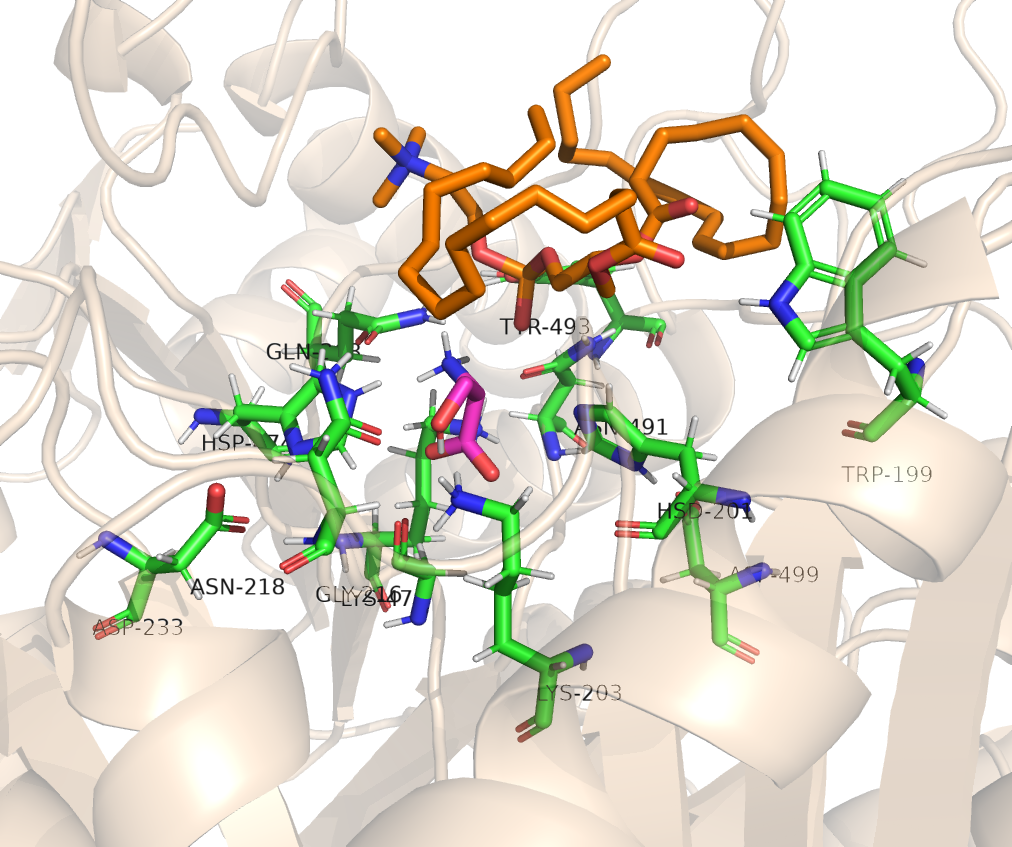

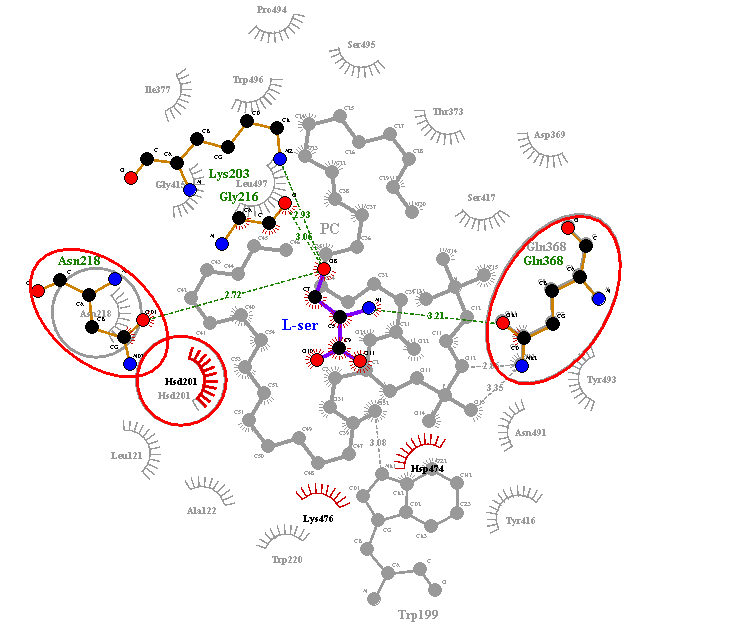

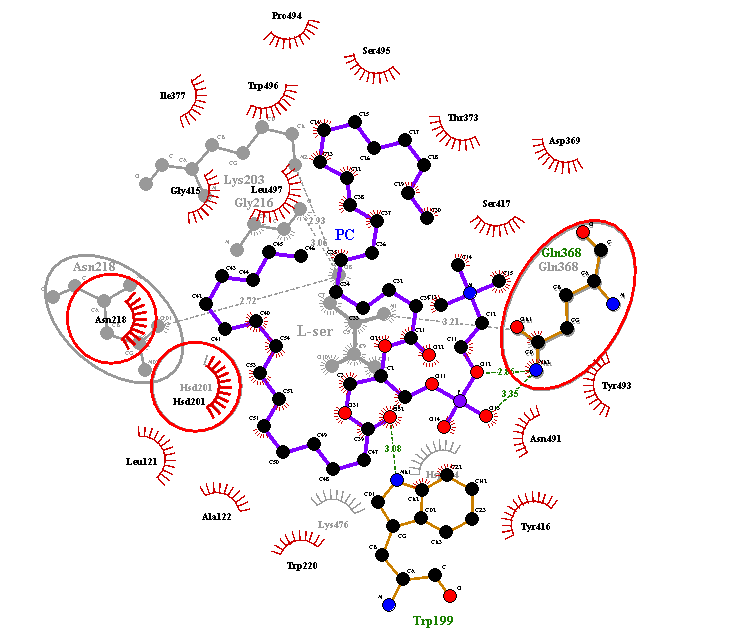

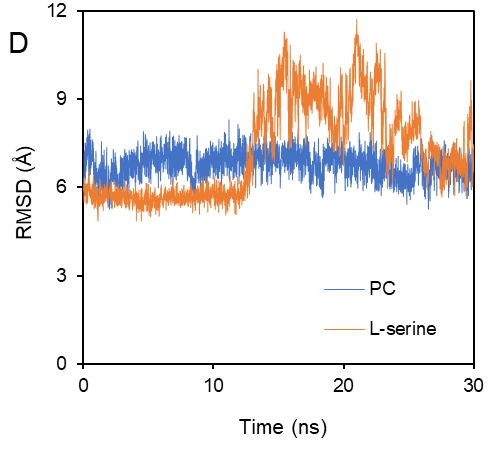


A

B

C

Until now only phosphoric acid and phosphatidic acid, not l‑serine or PC were reported to be co-crystalized with the PLD structures. Therefore, the molecular docking was combined with the MD simulation to confirm the crucial residues and their distances to the substrates inside the used PLD. As shown in Figure S2, substrates of l‑serine and PC were simultaneously docked into the active center of PLD, and, then, MD were employed to study the stability of this PLD-serine-PC (PLD-Ser-PC) complex. As shown in Figure S2D, the crucial distances between the active sites of PLD and its two substrates (PC and l‑serine) remained at 7 and 6 Å, respectively. However, the free diffusion process is significantly more difficult to reconstruct *in silico* than the dissociation of enzyme-ligands complex, due to the strong effect of solvents. Therefore, these values were doubled (12 and 10 Å) to judge whether these ligands were in the active pocket. Similar approaches were reported by previous works.

**Figure S3.** Kinetic behaviors of ligands, including the time evolution of RMSD, diffusional trajectories (only molecules bound in the active pocket were shown), binding time of substrates (each color represents one substrate molecule) in the diethyl ether-water system.

L-serine

PC

L-serine

PC

PC

PC

L-serine

L-serine

**Figure S4.** Kinetic behaviors of ligands, including the time evolution of RMSD, diffusional trajectories (only molecules bound in the active pocket were shown), binding time of substrates (each color represents one substrate molecule in the coconut-water system.

****The missing figure mean that no ligand was found in the active pocket.

L-serine

PC

L-serine

PC

L-serine

PC

PC

L-serine

**Figure S5.** Kinetic behaviors of ligands, including the time evolution of RMSD, diffusional trajectories (only molecules bound in the active pocket were shown), binding time of substrates (each color represents one substrate molecule) in the olive oil-water systems.

****The missing figure mean that no ligand was found in the active pocket.

L-serine

PC

L-serine

PC

PC

L-serine

PC

L-serine

**Figure S6.** Substrates diffusions in the oil-water systems. Many l‑serine molecules were located arround the active center of PLD, which prevented the entry of PC molecules. PC and l‑serine were represented by orange and magenta, respectively. Two crucial histidines (HSP) 201 and 474 were shown by green. All solvent molecules were hidden for an easier visualization. (A) coconut oil-water system; (B) olive oil-water.


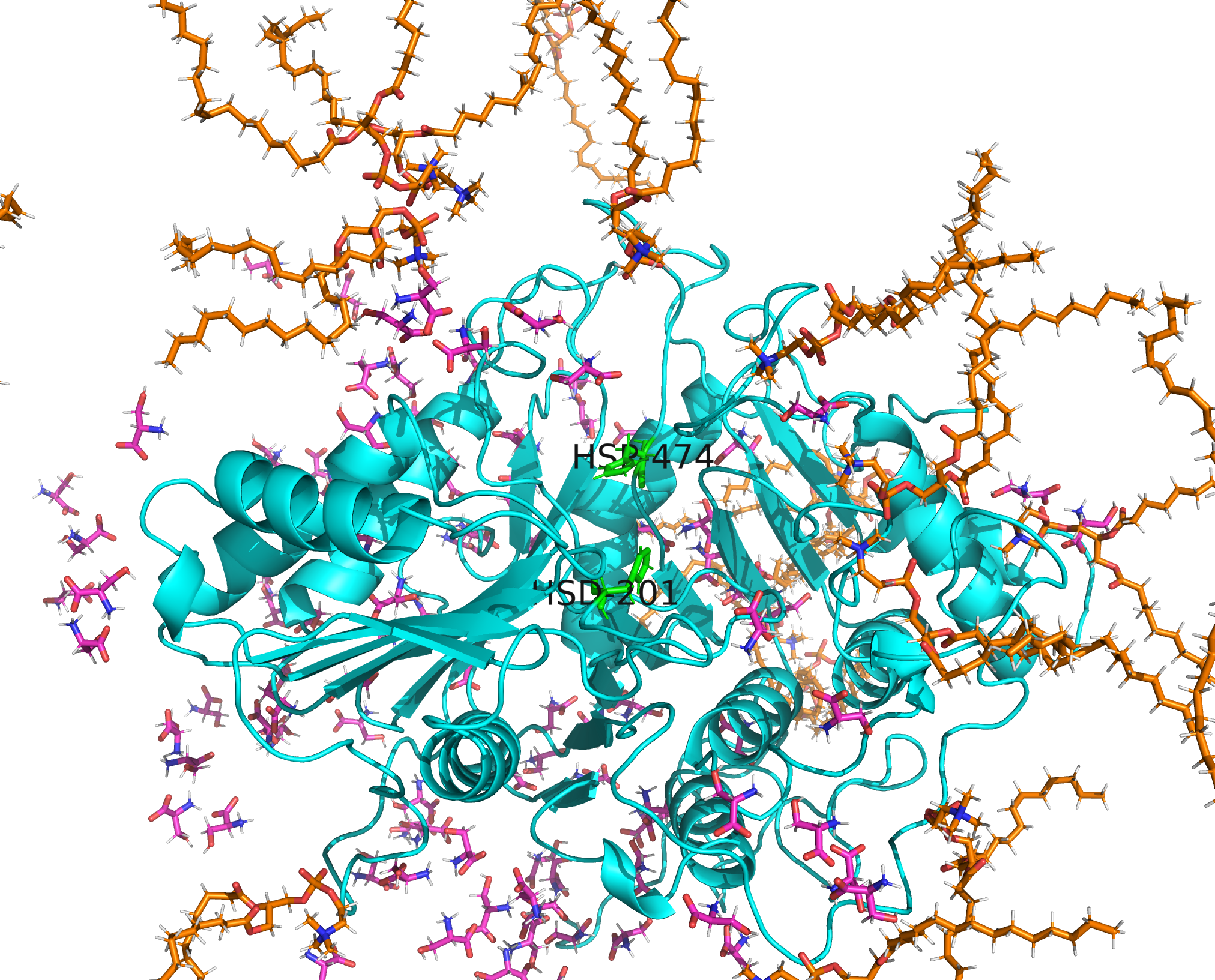

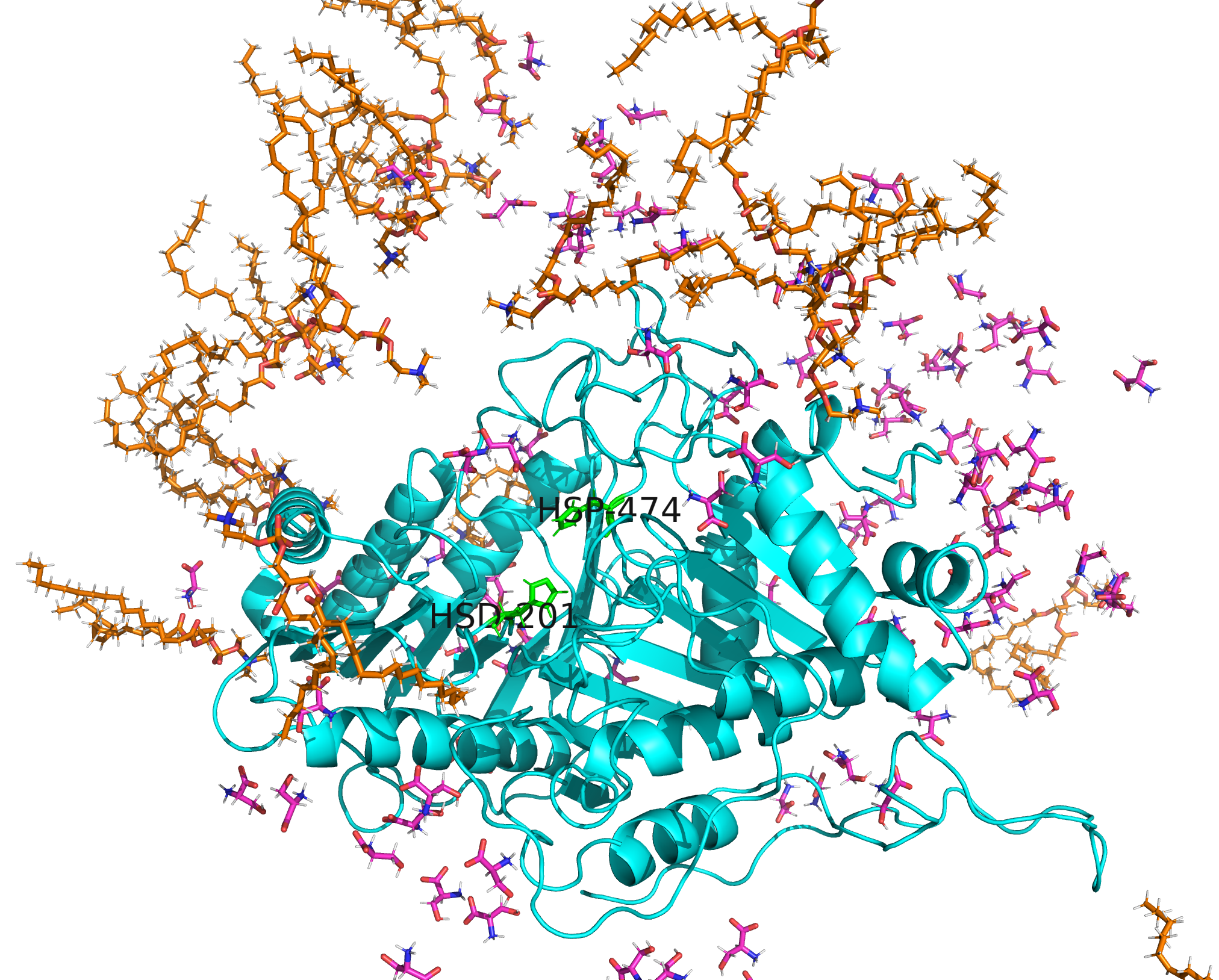


A

B

**Figure S7.** Water molecules within 10 Å from the active pocket of PLD in (A) diethyl ether-water; (B) coconut oil-water; (C) olive oil-water systems.


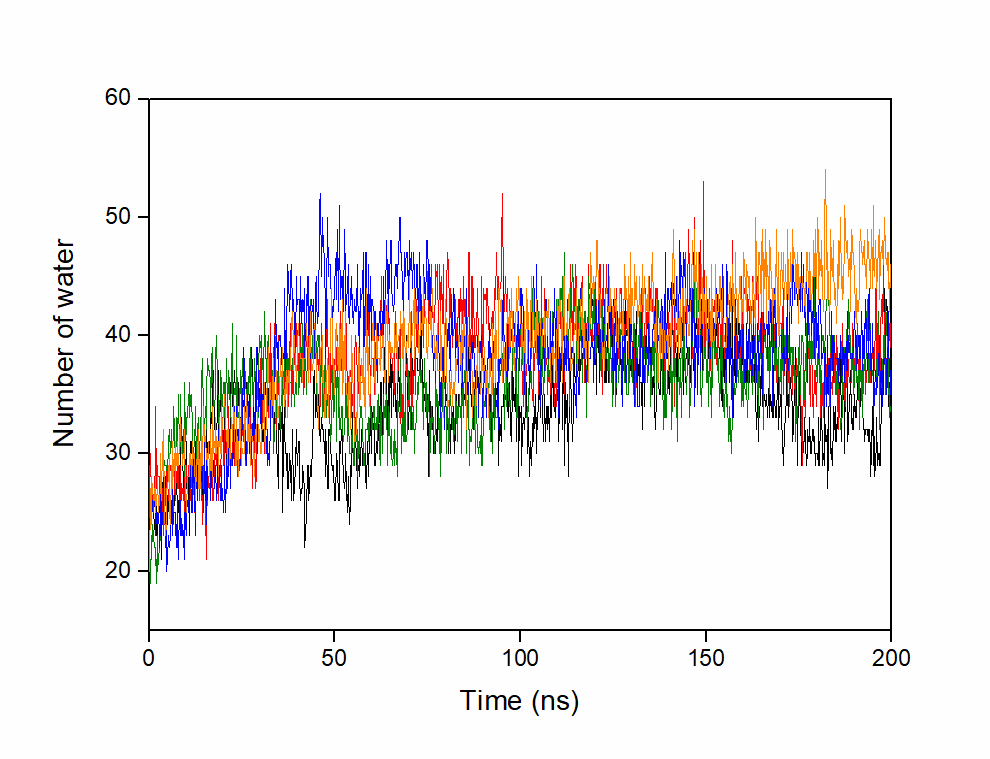


B


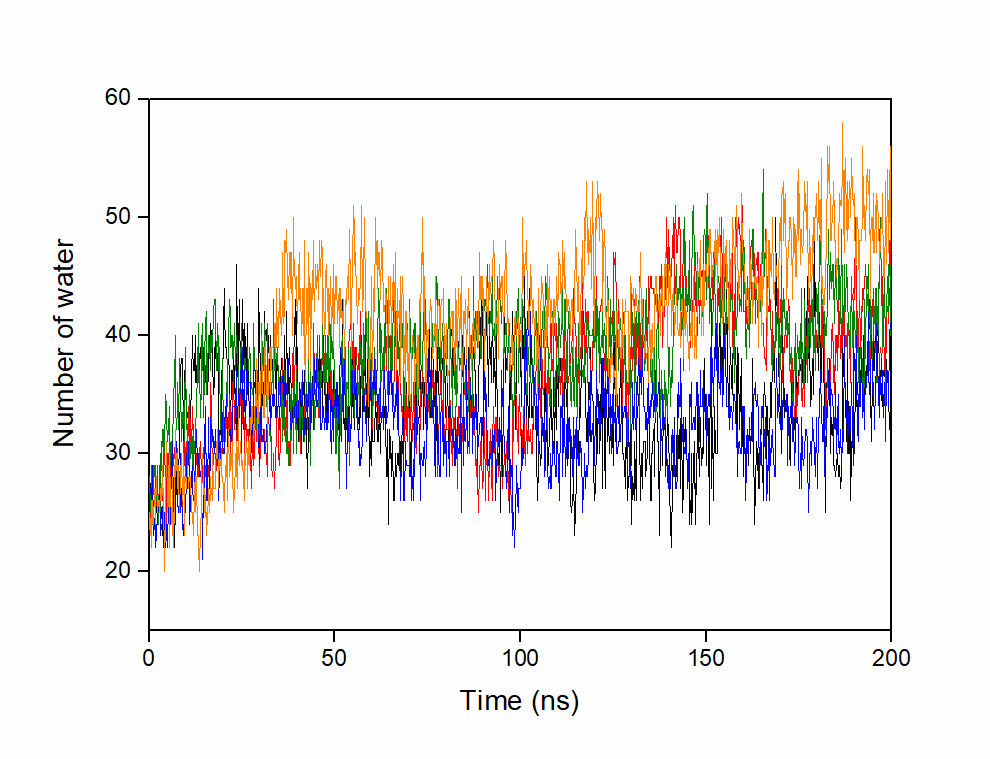


A


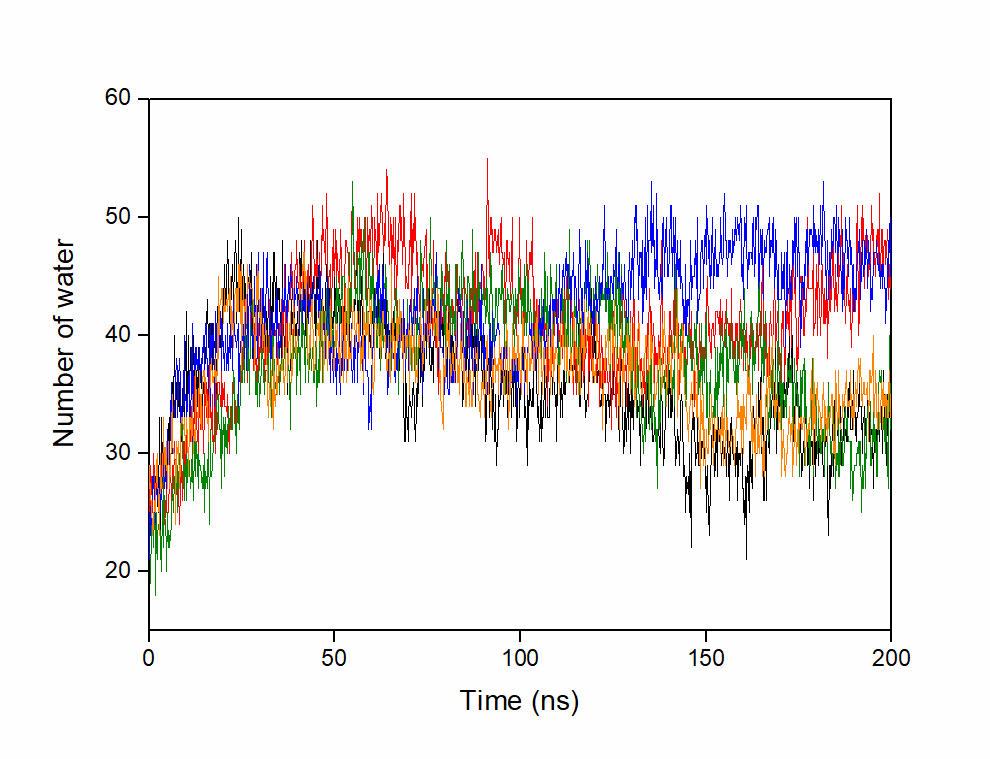


C

**Figure S8.** Stabilities of PLD-PS complex structures in (A) diethyl ether-water; (B) coconut oil-water; (C) olive oil-water systems. Time evolution of RMSD for the distance of P atom (PS) and N atom (PLD, H201) during 5 times of MD simulations. The PLD-PS complex structure was generated by the molecular docking. Then, this complex was put in three micro-units just like Figure 1. The dissociation of PS from the active center of PLD was evaluated in the presence of PC and L-serine molecules to simulate real reaction conditions. The PLD-PS complex structure was generated by the molecular docking firstly. Then, this complex was put in three micro-units just like Figure S3. The PLD-PS complex structure was generated by the molecular docking firstly. Then, this complex was put in three micro-units just like Figure S3. PC and L-serine molecules were also added to simulate real reaction conditions.


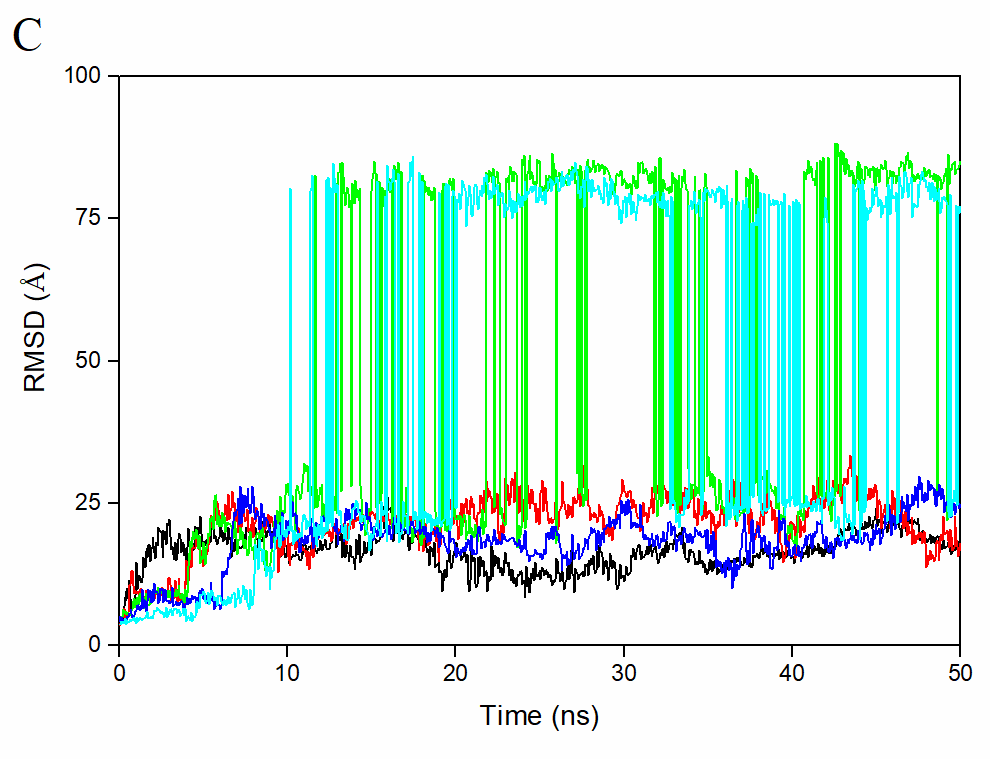

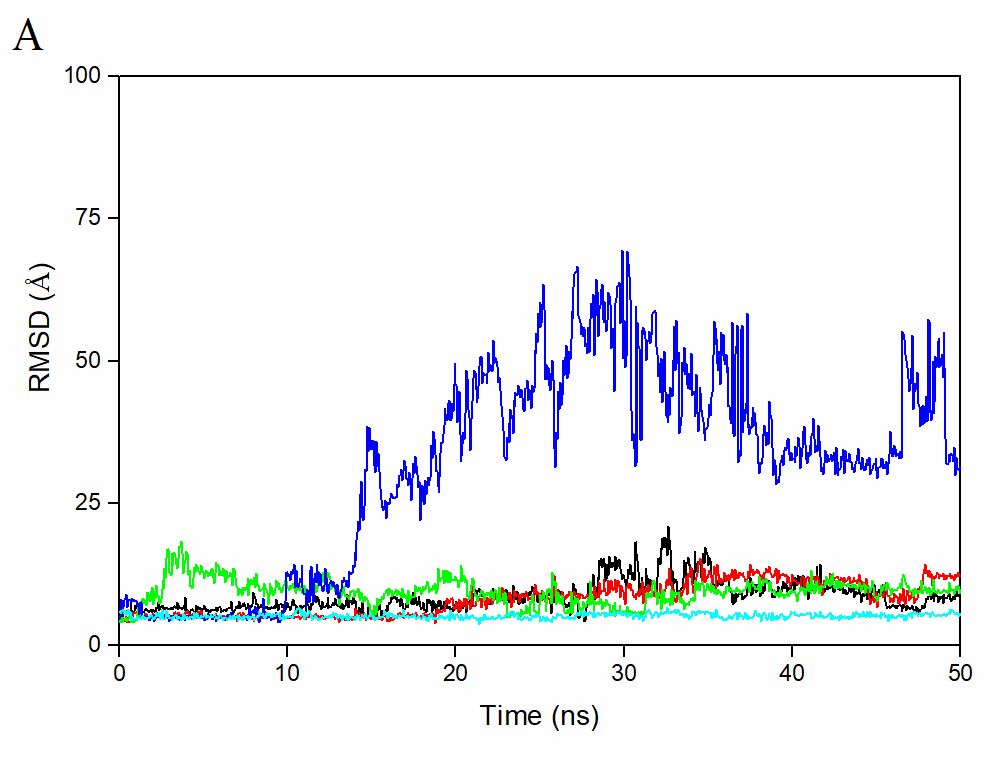

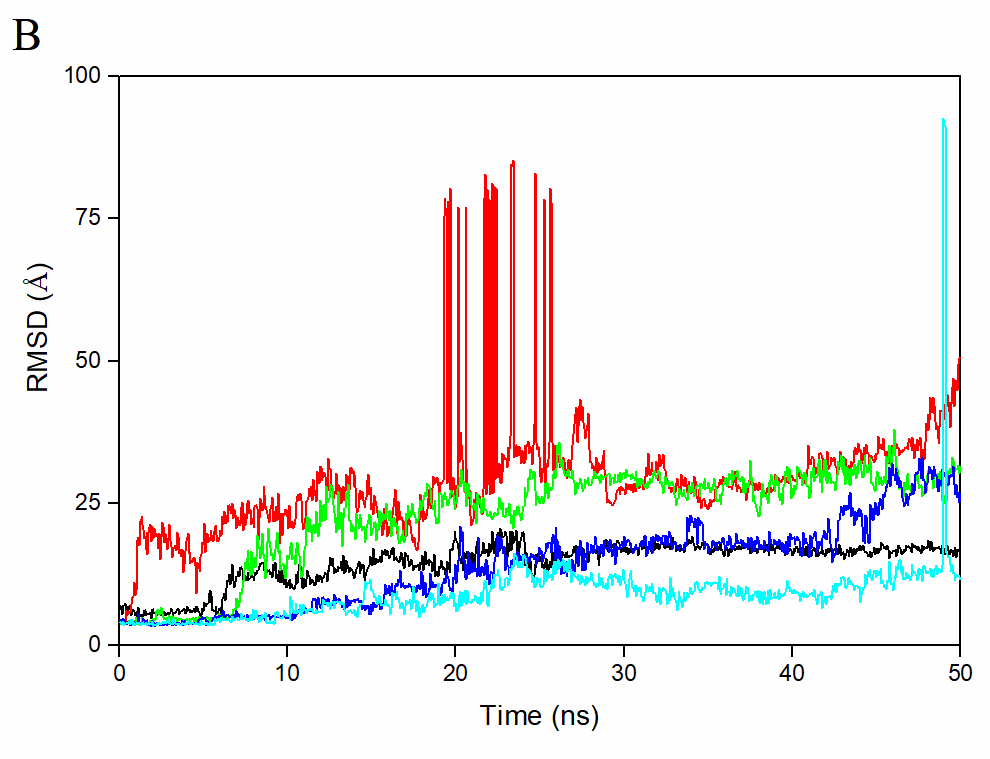


**Figure S9.** Scheme of diffusional channels of PLD. Four important loops were shown by different color. Two crucial histidines (H474 and H201) were represented as green color to only guide the location of active pocket of PLD. The diffusional channels existed between different loops.


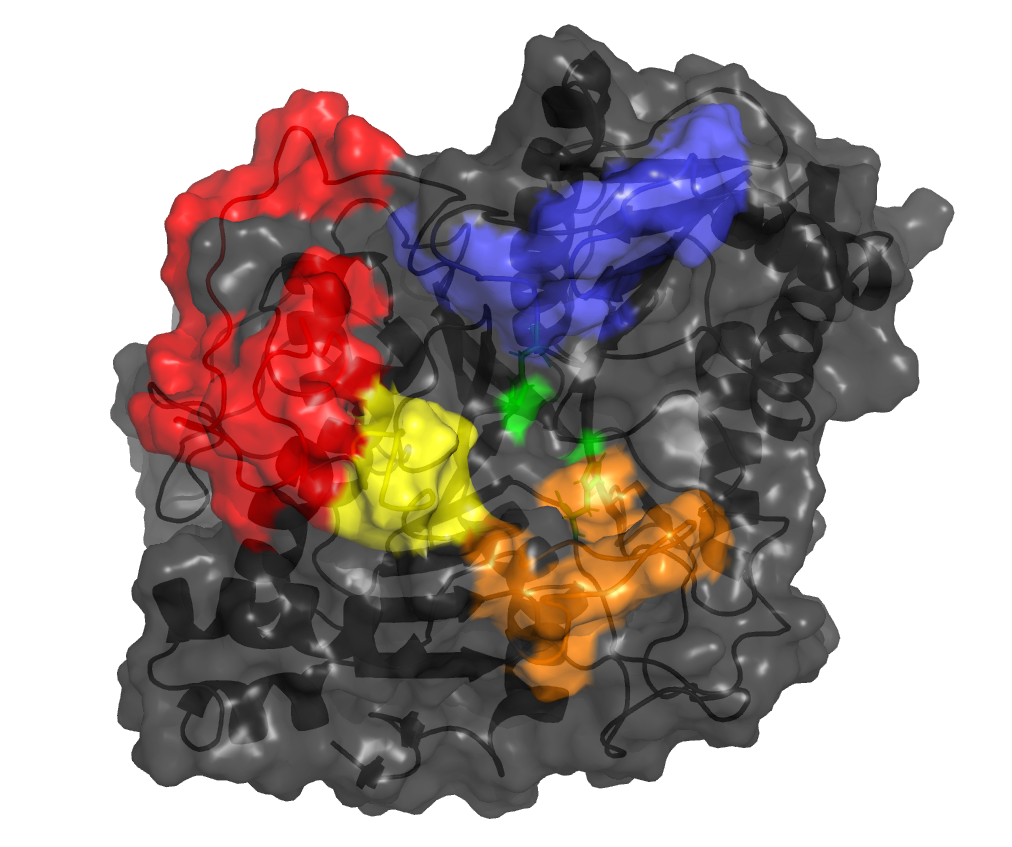


Loop Ⅱ

Loop Ⅰ

Loop Ⅲ

Loop Ⅳ

**Figure S10.** Microcapsules of coconut oil-PS (A) and olive oil-PS (B).


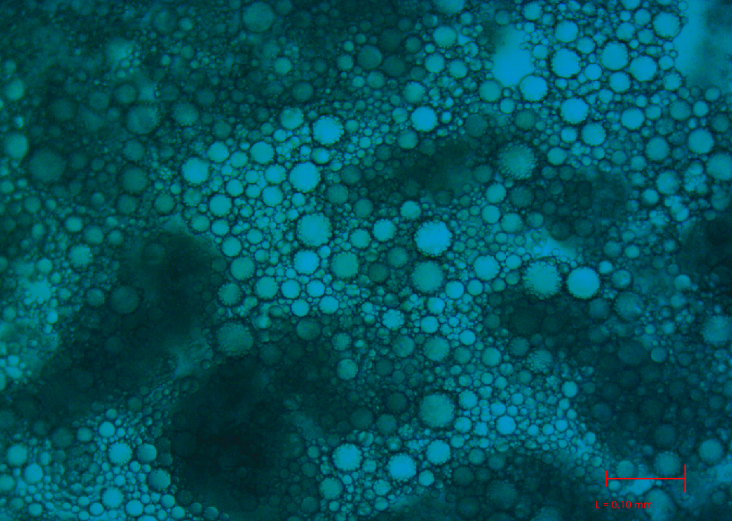


B


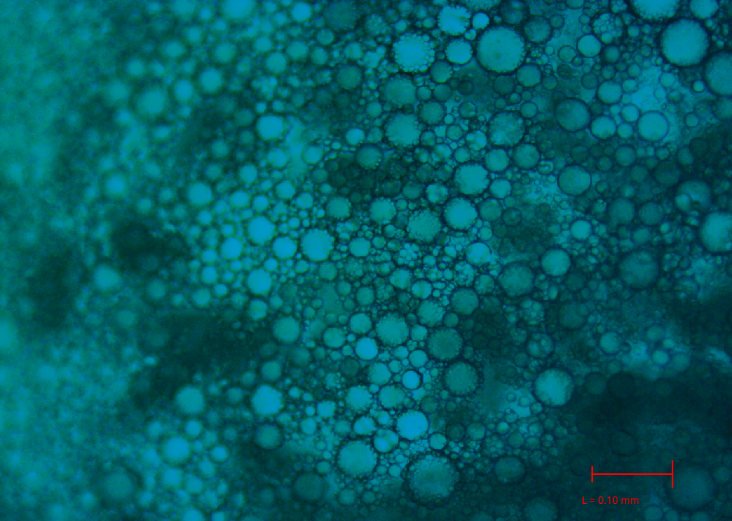


A
